# Nonstructural Protein NS80 Is Crucial in Recruiting Viral Components to Form Aquareoviral Factories

**DOI:** 10.1371/journal.pone.0063737

**Published:** 2013-05-06

**Authors:** Fei Ke, Li-Bo He, Qi-Ya Zhang

**Affiliations:** State Key Laboratory of Freshwater Ecology and Biotechnology, Institute of Hydrobiology, Chinese Academy of Sciences, Wuhan, China; Utah State University, United States of America

## Abstract

**Background:**

Replication and assembly of vertebrate reoviruses occur in specific intracellular compartments known as viral factories. Recently, NS88 and NS80, the nonstructural proteins from aquareoviruses, have been proposed to share common traits with µNS from orthoreoviruses, which are involved in the formation of viral factories.

**Methodology/Principal Findings:**

In this study, the NS80 characteristics and its interactions with other viral components were investigated. We observed that the NS80 structure ensured its self-aggregation and selective recruitment of viral proteins to viral factories like structures (VFLS). The minimum amino acids (aa) of NS80 required for VFLS formation included 193 aa at the C-terminal. However, this truncated protein only contained one aa coil and located in the nucleus. Its N-terminal residual regions, aa 1–55 and aa 55–85, were required for recruiting viral nonstructural protein NS38 and structural protein VP3, respectively. A conserved N-terminal region of NS38, which was responsible for the interaction with NS80, was also identified. Moreover, the minimal region of C-terminal residues, aa 506–742 (Δ505), required for NS80 self-aggregation in the cytoplasm, and aa 550–742 (Δ549), which are sufficient for recruiting viral structure proteins VP1, VP2, and VP4 were also identified.

**Conclusions/Significance:**

The present study shows detailed interactions between NS80 and NS38 or other viral proteins. Sequence and structure characteristics of NS80 ensures its self-aggregation to form VFLS (either in the cytoplasm or nucleus) and recruitment of viral structural or nonstructural proteins.

## Introduction

Viral factories (also termed viral inclusion bodies, viroplasm, or viromatrix) are specific intracellular matrices and physical scaffolds where multiple viral components participate in viral replication and assembly. The formation of viral factories is complex and involves a series of interactions among different types of viral components, including structural and nonstructural proteins. Many recent efforts have been dedicated to identify key factors and processes in the formation of viral factories [1]–[4].

Members of the family Reoviridae are known to replicate and assemble within the cytoplasmic viral factories. Reoviridae contains a large and diverse group of viruses with icosahedral symmetry but may appear spherical, and non-enveloped with one-, two-, or three-layered protein capsids surrounding the linear dsRNA segments of the viral genome. Viruses in this family are divided into 15 genera [5]. Although the aquareovirus genome (reoviruses that infects aquatic species such as fish, shellfish) comprises 11 dsRNA segments and that of orthoreovirus (reoviruses that infects mammals, birds, or reptiles) comprises 10 dsRNA segments, these two distinct members are considered the most closely related genera in Reoviridae based on multiple parameters [6], [7].

Aquareoviruses have been isolated from a wide variety of aquatic animals such as fish, molluscs, finfish, and crustaceans [6], [8], [9]. Viruses including reovirus, iridovirus, and rhabdovirus can all cause diseases in aquatic organisms. These viruses produce viral factories (they can be either cytoplasmic or nuclear), where the inclusions with crystalline arrays of virus particles are often visible [8], [10], [11]. The related and unrelated viruses in aquatic organisms have been reported to mediate the building of viral factories that function in virus replication [12]–[14]. These viruses infect and destroy host cells of aquatic organisms. The molecular mechanisms for infection and damage in aquatic species used by viral pathogens are diverse [9], [15]–[21]. Reoviruses have been studied as model viruses to determine the structure of viral particles, mechanisms of virus replication, pathogenesis, and virus-host interactions [2], [22]–[28].

Recently, NS88, a nonstructural protein of turbot *Scophthalmus maximus* reovirus (SMReV) was found to share notable homology with orthoreovirus µNS. Another nonstructural protein of SMReV, NS38, has been predicted to be involved in the formation of viral inclusion bodies with NS88 [9]. In addition, NS80, another nonstructural protein of aquareovirus has also been recognized as similar to SMReV NS88 and orthoreovirus µNS that play common roles in the formation of viral factories like structures (VFLS) [29]. However, a lack of detailed investigation for the expression features and protein recruitment of NS88, NS80, and their homologs hampers our understanding on the formation of aquareoviral factories and the virus replication mechanism.

In the present study, the central role of NS80 in viral factory formation was investigated. Based on the analysis of the sequence structure, expression products (full-length or truncated), and its interactions with other viral proteins, it is possible to elucidate the role of NS80. As a nonstructural protein, it showed specific subcellular localization and recruited viral proteins via certain aa sequences.

## Results

### C-terminal residues of NS80 affect the formation of viral factories like structures

**Figure 1:**
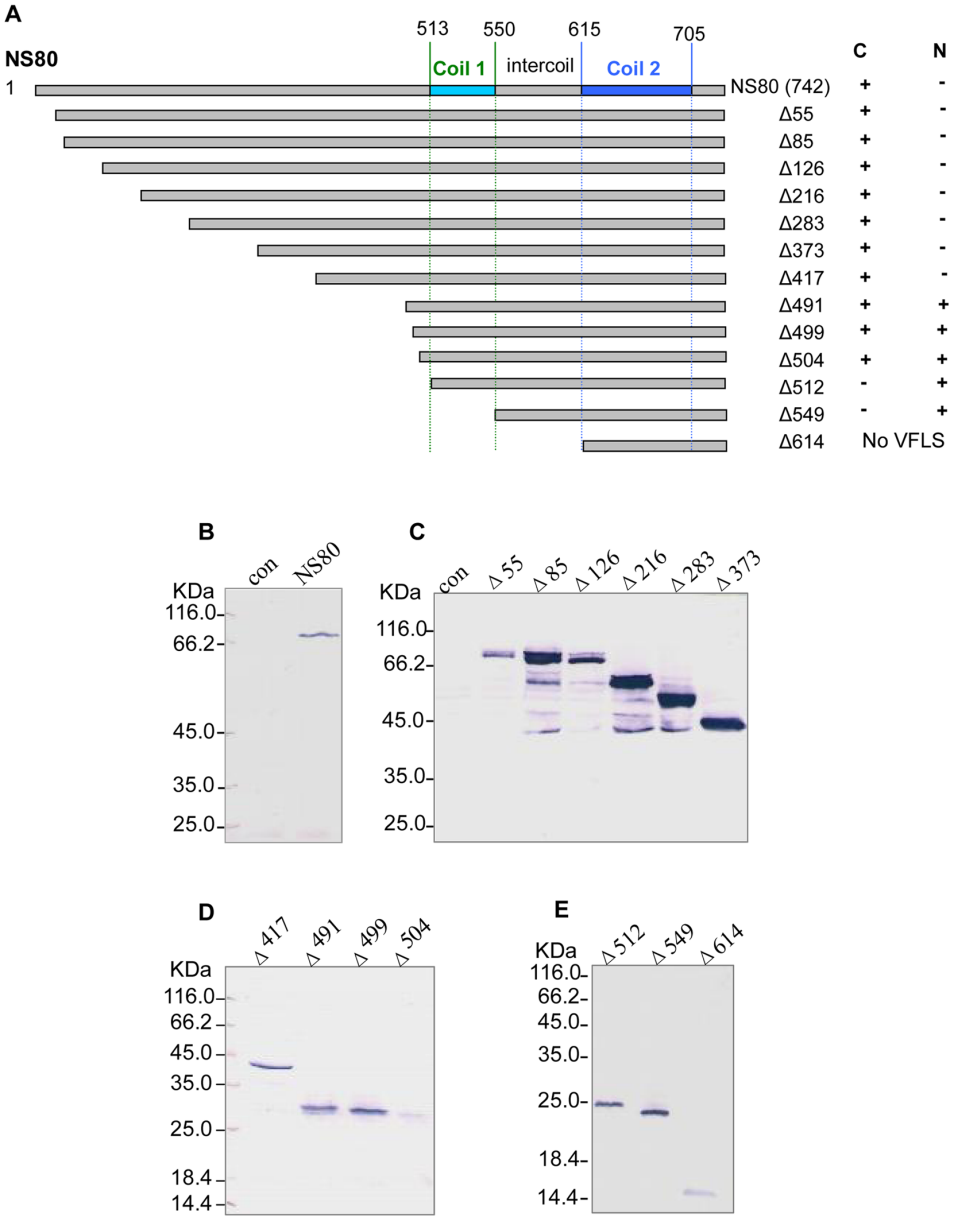
Detection of full-length or truncated NS80 by Western blotting. A. Diagram of NS80 N-terminal truncations. The two predicted coil and intercoil regions are indicated. Full-length (742) or truncated fragments (Δ55–Δ614) were fused with FLAG tags and then inserted into the pcDNA3.1 vector. C, cytoplasmic; N, nucleus. B, C, D, E. Western blot analysis of expression products of full-length and truncated NS80 in cells transfected with anti-FLAG antibodies. Full-length or N-terminal truncated NS80 is indicated at the top of the figure. The detections (B, C, and D) were carried out using 12% SDS-PAGE and the detections (E) were carried out using 15% SDS-PAGE.

NS80 contained two coils (residues 513–550 and 615–705, Fig. 1A) according to predictions made by the coils program [30]. To identify the NS80 domains responsible for VFLS formation, the NS80 N-terminal was deleted in a stepwise manner (Fig. 1A), fused with FLAG tag, and then inserted into pcDNA3.1 vector.

**Figure 2:**
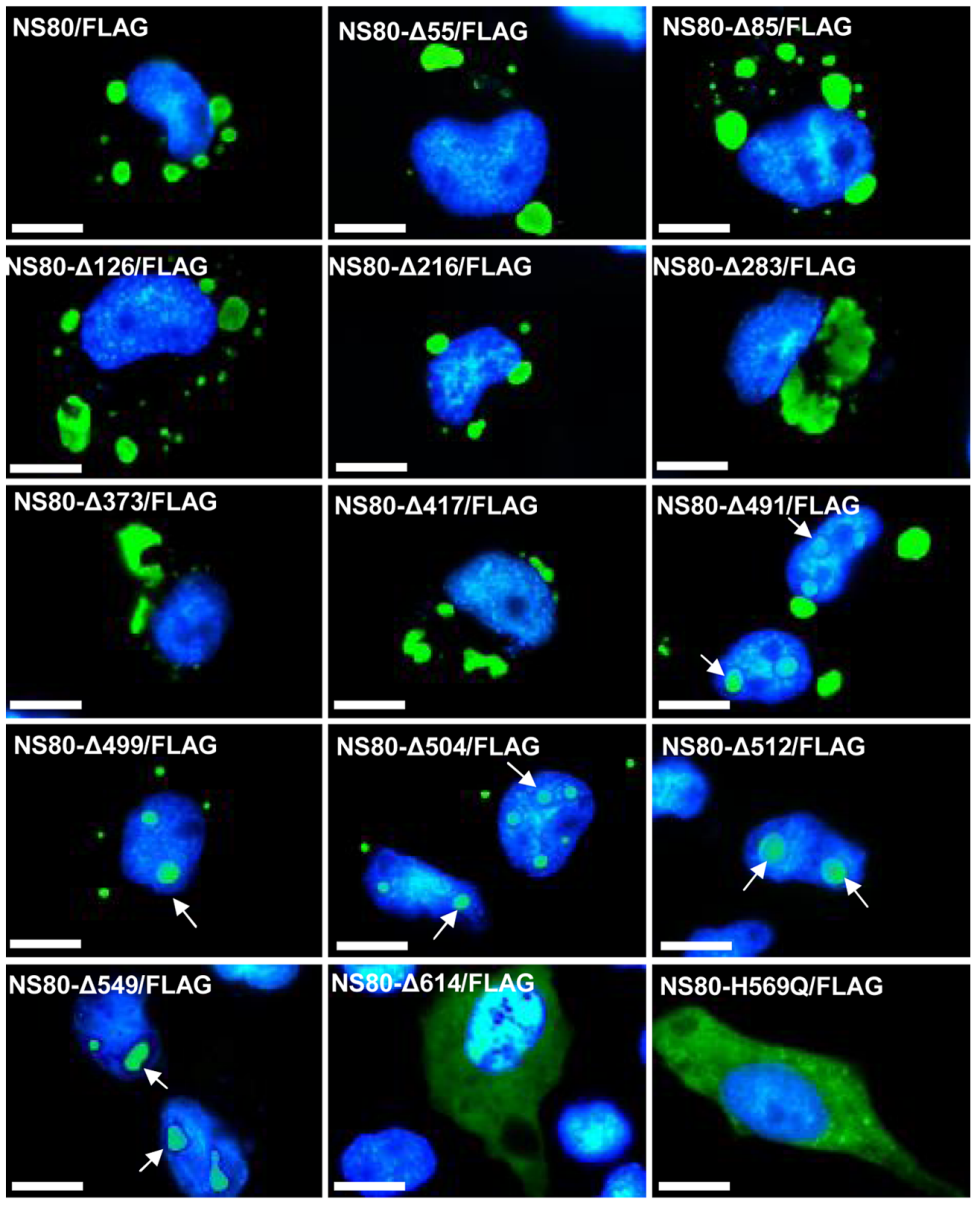
Subcellular distribution of full-length, truncated (Δ55–Δ614), or mutated (H569Q) NS80. Green fluorescence of NS80 truncations were distributed in the cytoplasm (NS80, Δ55–Δ417), gradually localized in the nucleus (Δ491–Δ549), or diffusely distributed in whole cells (Δ614 and H569Q). Blue fluorescence shows the nuclei stained by Hoechst 33342. Truncated NS80-FLAG proteins located in the nucleus are indicated with white arrows. Bar = 20 μm.

The recombinant plasmids were separately transfected into the GCO cells. The expression of each construct was identified by Western blot analysis (Fig. 1B, C, D, and E). The capacities of these constructs to form globular inclusions were analyzed by immunofluorescence with anti-FLAG antibodies. The expression of NS80-FLAG resulted in the formation of globular inclusions (NS80/FLAG in Fig. 2), which were similar to those found in viral factories. Deletion of the 216 N-terminal residues had no adverse effect on the formation of inclusions. However, the inclusions formed by NS80 truncations lacking N-terminal 283 and 373 residues (NS80-Δ283/FLAG and NS80-Δ373/FLAG in Fig. 2) formed inclusions that were less spherical and smooth compared with those formed by the intact NS80.

VFLS formed in the cytoplasm during expression of the truncated mutant NS80 (418–742) (NS80-Δ417/FLAG in Fig. 2). Interestingly, globular inclusions formed by both the truncations NS80 (513–742) (NS80-Δ512/FLAG in Fig. 2), which contained the two predicted coils, and NS80 (550–742) (NS80-Δ549/FLAG in Fig. 2), lacking the first coil regions, were located in the nucleus rather than in the cytoplasm. In addition, globular inclusions formed by NS80 truncations lacking N-terminal 491, 499, or 504 residues were located in the entire cell, including the cytoplasm and nucleus. Expression of truncated NS80 (615–742) (NS80-Δ614/FLAG in Fig. 2) containing only one predicted coil showed a diffused distribution in cells. These results suggested that the 193 C-terminal residues comprising residues 550–742 compose the minimal region required for the formation of globular inclusions. However, the 512 NS80 N-terminal residues could affect the cytoplasmic location of inclusions, and the intercoil regions (aa 550–615 of NS80) play an important role in protein aggregation and nuclear localization.

In addition, the protein (NS80-H569Q) with a histidine mutation (replaced by glutamine) at position 569 could not form inclusions (NS80-H569Q/FLAG in Fig. 2). Histidine is conserved in the NS80 intercoil region and its homologs in aquareoviruses, suggesting that it may play an important role in VFLS formation.

### NS80 recruits nonstructural proteins to viral factories like structures

**Figure 3:**
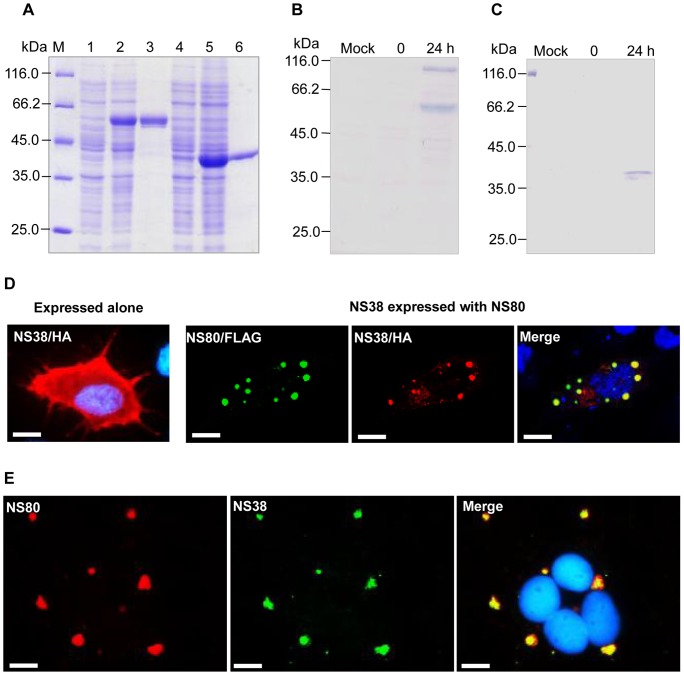
NS80 colocalized with NS38 in transfected and infected cells. A. Prokaryotic expression of the NS80 and NS38 fusion proteins. M: protein molecular weight marker; lane 1: bacterial lysate containing pET32a-NS80 without IPTG induction; lane 2: bacterial lysate containing pET32a-NS80 with IPTG induction; lane 3: the purified NS80 fusion protein; lane 4: bacterial lysate containing pET28a-NS38 without IPTG induction; lane 5: bacterial lysate containing pET28a-NS38 with IPTG induction; lane 6: the purified NS38 protein. B. Western blot analysis of NS80 expression in infected cells. Two obvious protein bands were detected at 24 h p.i. using anti-NS80 rabbit serum. C. Western blotting analysis of NS38 expression in infected cells. D. In the transfected cells, NS38 (red) is diffusely distributed when expressed alone. However, when NS38 is coexpressed with NS80 (green), they colocalized (yellow). Blue fluorescence indicates the nuclei stained by Hoechst 33342. Bar  = 20 μm. E. In infected cells, NS80 (red) and NS38 (green) colocalized in viral factories. Blue fluorescence shows the nuclei stained by Hoechst 33342. Bar  = 20 μm.

The nonstructural protein NS38 contains 352 aa with a molecular mass of 38 kDa and is encoded by the S9 genome segment. It has a high homology with the mammalian orthoreovirus (MRV) σNS with respect to its secondary structure and hydrophobicity. Other viral proteins are also required for the formation and function of viral factories. Interactions between NS80 and NS38 were analyzed. When pcDNA3.1/NS80-FLAG and pcDNA3.1/NS38-HA were transfected in GCO cells respectively, NS80 assembled in the cytoplasm to form VFLS as indicated by green fluorescence (Fig. 2), and NS38 was diffusely distributed in the cytoplasm as indicated by red fluorescence (Fig. 3D). When these two plasmids were cotransfected, NS80 and NS38 colocalized in VFLS in the cytoplasm (presented as yellow in the merged file in Fig. 3D), indicating that NS80 assembled in the cytoplasm and recruited NS38.

Distribution of NS80 and NS38 in virus infected cells was examined by an immunofluorescence assay. NS80 and NS38 were individually expressed in *Escherichia coli* BL21 (DE3) and purified for antibody preparation (Fig. 3A, B, and C). In the immunofluorescence assay, NS80 was detected by anti-NS80 rabbit serum with Alex fluor 546-conjugated goat anti-rabbit secondary antibodies (red fluorescence) and NS38 was detected by anti-NS38 mouse serum with Alex fluor 488-conjugated goat anti-mouse secondary antibodies (green fluorescence). As shown in Fig. 3E, NS80 was aggregated and colocalized with NS38 (presented as yellow in the merged file). The nuclei of infected cells were aggregated, a typical phenomenon of aquareovirus infection.

**Figure 4:**
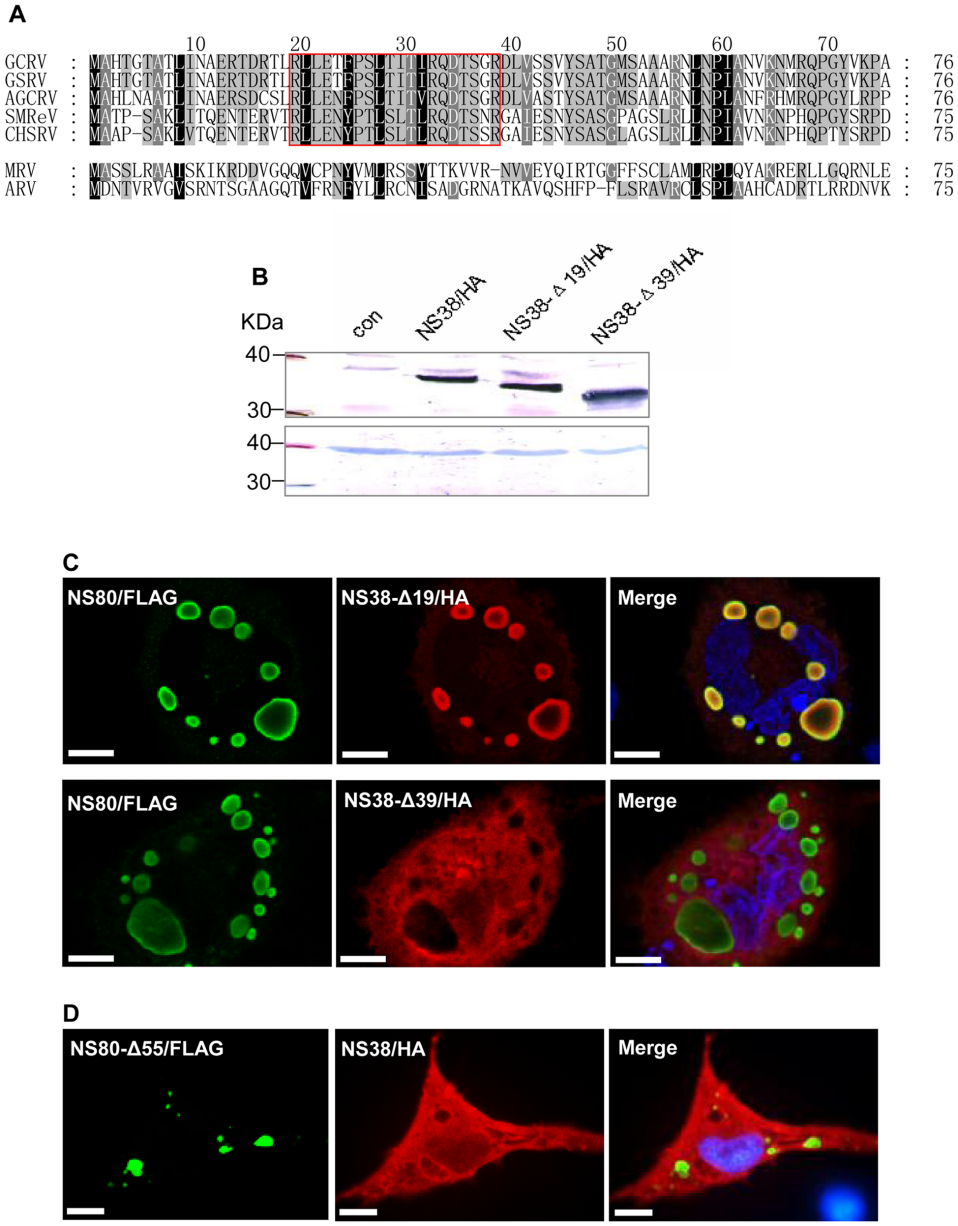
Amino acid sequence comparison of NS38 from aquareoviruses and orthoreoviruses, and immunofluorescence analysis of key sequences in interactions between NS80 and NS38. A. Amino acids alignment of N-terminal sequences of NS38 from five aquareoviruses (GCRV, CSRV, AGCRV, SMReV and CHSRV) and its homologs belonging to orthoreoviruses (σNS in MRV or ARV). The black shaded regions indicate highly conserved residues in all viruses, while the gray shaded regions are partially conserved residues with more than 80% identity. The key region involved in protein interactions is boxed (aa 20–39 of GCRV). B. Western blot analysis of expression products of full-length and truncated NS38 in transfected cells C. Immunofluorescence analysis of NS80 coexpressed with NS38 N-terminal truncations in transfected cells. NS38-Δ19 (red) colocalized with NS80 (green) but NS38-Δ39 (red) was not colocalized with NS80 (green), which indicated that the NS38 conserved residues, 20–39, were responsible for colocalization with NS80. The nucleus was stained by Hoechst 33342 and presented as blue. Bar  = 20 μm. D. Immunofluorescence analysis of NS38 coexpressed with NS80 N-terminal truncations in transfected cells. NS80-Δ55 (green) was not colocalized with NS38 (red). The nucleus was stained by Hoechst 33342 and presented as blue. N-terminal residues, 1–55, of NS80 were important for interactions with NS38. Bar  = 20 μm.

Results of the above studies revealed that NS80 could recruit NS38. aa sequence comparisons showed that a conserved region existed in the NS38 N-terminal (residues 20–39 of NS38) and its homologs (Fig. 4A). To determine the roles of this conserved region in interactions between NS80 and NS38, the NS38 N-terminal was deleted in a stepwise manner. The expression of truncated NS38 was detected by Western blotting (Fig. 4B). When NS80-FLAG was coexpressed with NS38-Δ19-HA (1–19 aa deleted), they colocalized in the cytoplasm (Fig. 4C), but NS80-FLAG could not colocalize with NS38-Δ39-HA (1–39 aa deleted) when they were coexpressed (Fig. 4C), indicating that the conserved 20–39 NS38 residues are important for interactions between NS38 and NS80.

Full length NS80-FLAG could recruit NS38-HA (Fig. 3D), but NS80-Δ55-FLAG, which had truncations of residues 1–55 could not colocalize with NS38-HA (Fig. 4D), suggesting that the 1–55 NS80 residues are responsible for interactions with NS38.

### NS80 recruits structural proteins to viral factories like structures

**Figure 5:**
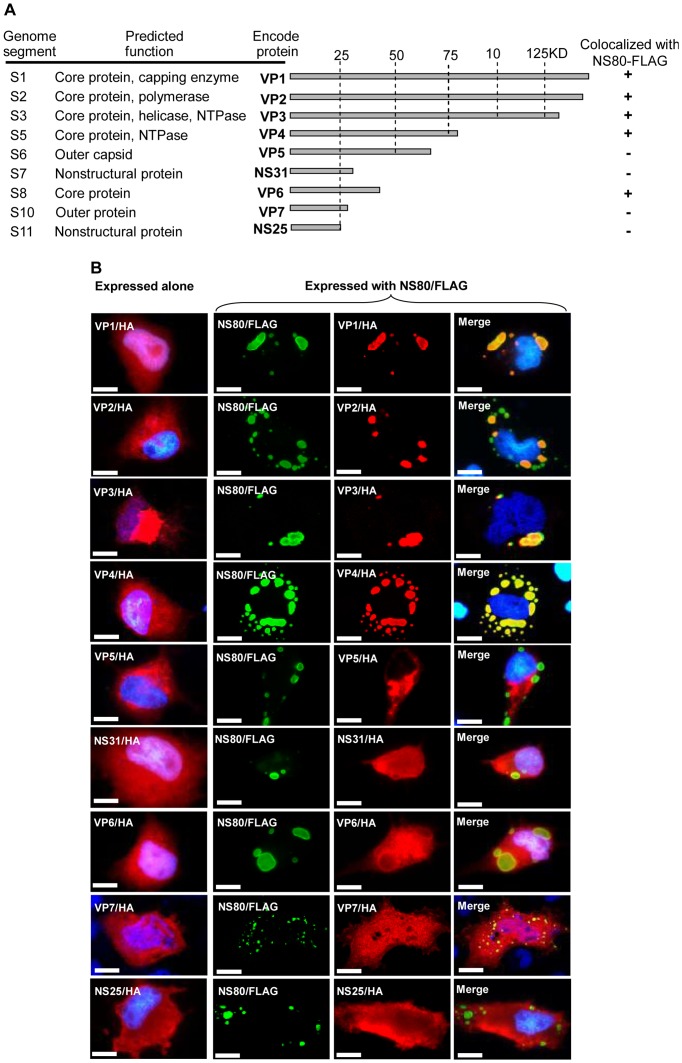
Diagram, summary, and immunofluorescence analysis of interactions between NS80 and the other nine viral proteins. A. Diagram and summary of interactions between NS80 and the nine viral proteins. “+” indicates that two proteins colocalized, and “−” indicates that two proteins were not colocalized. B. Immunofluorescence analysis of the nine viral proteins when expressed alone or with NS80. The nine viral proteins were expressed alone (lane on the left of the figure) or coexpressed with NS80 (the three lanes on the right of the figure). Green fluorescence indicated NS80. Red fluorescence indicated the other nine viral proteins. The nucleus was stained by Hoechst 33342 and presented as blue. NS80 colocalized with VP1, VP2, VP3, and VP4, and NS80 weakly colocalized with VP6. Bar  = 20 μm.

Viral factories are known as the place where viruses replicate and assemble. For this reason, viral factories interact not only with nonstructural proteins but also with structural proteins. The 11 GCRV genome segments encode 12 proteins. It has been known that NS80 could interact with NS38, but whether it could interact with the other proteins remains unclear. To identify other GCRV proteins that could interact with NS80, two tags, FLAG and HA, were used for visual localization of viral proteins. Each viral protein (except NS80) was fused with the N-terminal of HA and the resulted plasmid was cotransfected with pcDNA3.1/NS80-FLAG (Fig. 5A). Results are summarized in Fig. 5A.

Among the 10 viral proteins examined, NS80 (green fluorescence) strongly colocalized with VP1, VP2, VP3, VP4, and NS38 recombinant protein (red fluorescence; Fig. 5B and Fig. 3D), weakly colocalized with VP6 (Fig. 5B) but not with the other viral proteins (Fig. 5B).

To determine the NS80 aa regions responsible for interactions with these four proteins (VP1, VP2, VP3, and VP4), NS80 N-terminals were gradually deleted and the C-terminals were fused with the FLAG tag. These truncated recombinant proteins were coexpressed with VP1-HA, VP2-HA, VP3-HA, and VP4-HA, respectively.

**Figure 6:**
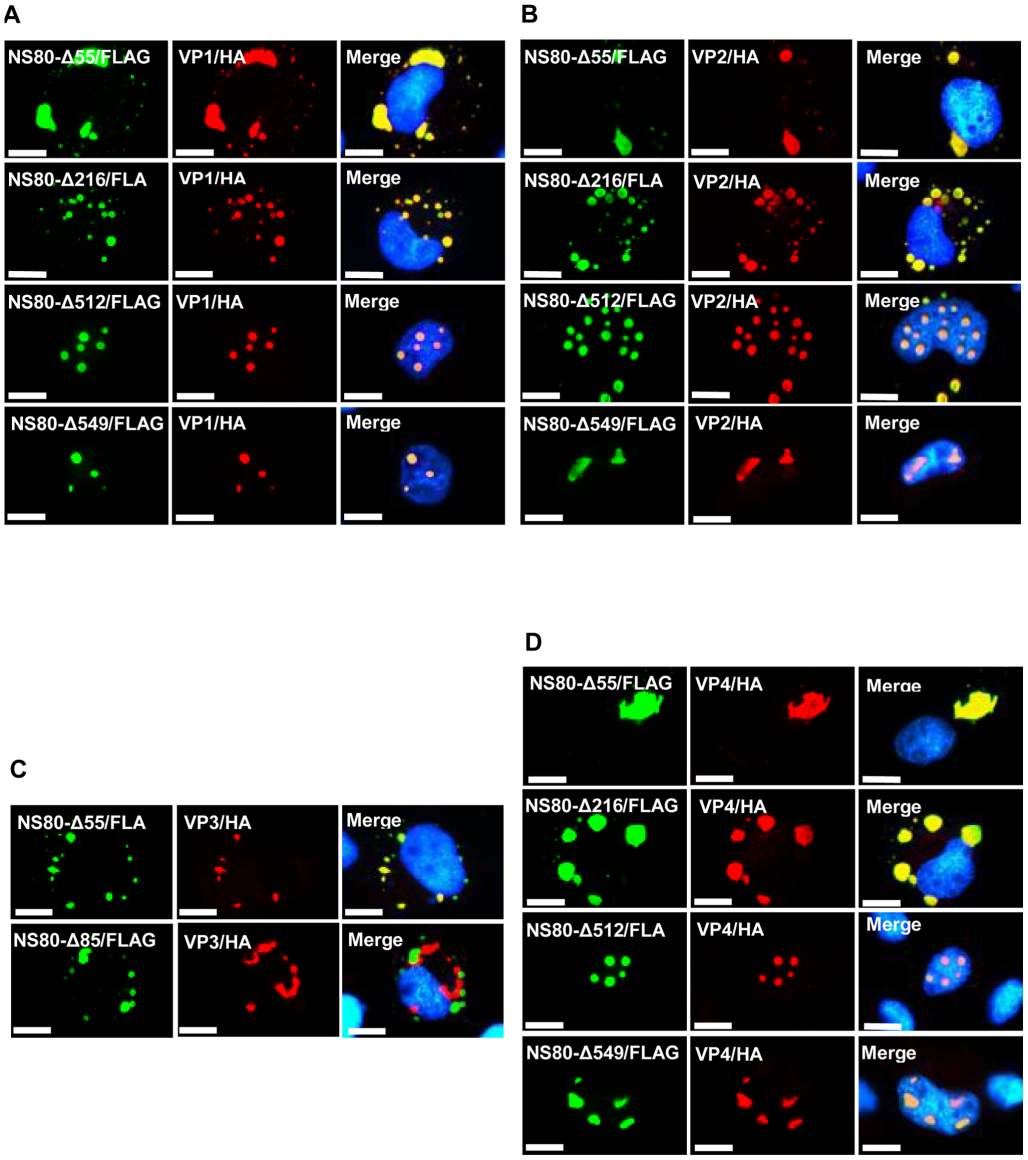
Immunofluorescence analysis of the distributions of N-terminal truncated NS80 and VP1, VP2, VP3, or VP4 when coexpressed. For all the pictures, green fluorescence indicated N-terminal truncated NS80, and red fluorescence indicated VP1, VP2, VP3, or VP4. The nucleus was stained by Hoechst 33342 and presented as blue. Colocalized proteins were presented as yellow in merged figures. Bar  = 20 μm. A. Immunofluorescence analysis of coexpression of VP1 and N-terminal truncated NS80. NS80-Δ55 and NS80-Δ216 colocalized with VP1 in the cytoplasm, but NS80-Δ512 and NS80-Δ549 colocalized with VP1-HA in the nucleus. B. Immunofluorescence analysis of the coexpressed VP2 and N-terminal truncated NS80. NS80-Δ55 and NS80-Δ216 colocalized with VP2 in the cytoplasm but NS80-Δ512 (partially) and NS80-Δ549 colocalized with VP2 in the nucleus. C. Immunofluorescence analysis of coexpressed VP3 and N-terminal truncated NS80. NS80-Δ55, not NS80-Δ85, colocalized with VP3 in the cytoplasm. D. Immunofluorescence analysis of coexpressed VP4 and N-terminal truncated NS80. NS80-Δ55 and NS80-Δ216 colocalized with VP4 in the cytoplasm, but NS80-Δ512 and NS80-Δ549 colocalized with VP4 in the nucleus.

A similar phenomenon was observed when NS80 N-terminal truncations were coexpressed with VP1, VP2, or VP4. NS80-Δ55-FLAG, NS80-Δ216-FLAG, NS80-Δ512-FLAG, and NS80-Δ549-FLAG colocalized with VP1 (Fig. 6A), VP2 (Fig. 6B), or VP4 (Fig. 6D). These results indicated that the residues responsible for the interactions with VP1, VP2, or VP4 localize in the 550–742 C-terminal residues. Interestingly, NS80-Δ512-FLAG and NS80-Δ549-FLAG recruited VP1, VP2, or VP4 into the nucleus and formed inclusions (Fig. 6A, B and D).

When VP3 recombinant protein was coexpressed with NS80-Δ55-FLAG in the cells, they colocalized in the cytoplasm (Fig. 6C), but NS80-Δ85-FLAG did not colocalize with VP3 when they were coexpressed (Fig. 6C), suggesting that 56–85 NS80 residues are responsible for the interactions between NS80 and VP3.

## Discussion

Different from the previous report on other reoviruses, the minimal NS80 region essential for aggregation only contained one intercoil and one coil (residues 550–742 of NS80, Fig. 2 and Table 1). It has been reported that the two coil regions of μNS are necessary for MRV viral factories formation [31]–[33]. It is also indicated as the difference between aquareovirus and other reovirus. Expression of NS80(492–742), NS80(500–742), NS80(505–742), and NS80(513–742) containing two coils and the intercoil localized into the nucleus, whereas NS80(418–742) localized in the cytoplasm. Perhaps the 418–491 NS80 residue region is important for localization in the cytoplasm. Compared with NS80(550–742), NS80(615–742) was diffusely distributed in the cytoplasm and nucleus, which indicated that the intercoil region (aa 550–615) plays a vital role in NS80 aggregation and localization. Several aspects may account for this difference in localization: (i) full-length NS80 could interact directly or indirectly with cytoplasmic compartments with its N-terminal sequence, which is responsible for cytoplasmic localization. Although the NS80 C-terminal could interact with nuclear compartments, its interaction was weaker than that of the N-terminal. It has been reported that in MRV (strain of T1L), μNS could colocalize with μ2 and then interact with microtubules in cytoplasm with the help of μ2 [34]. (ii) Besides the NS80 organizational role in viral factories formation, it may also have functions in the regulation of cellular response to infection (such as immunoregulation) by interacting with nuclear components. MRV (strain T1L) μ2, which has nuclear distribution, inhibits interferon signaling by accumulation of interferon regulatory factor 9 in the nucleus [35]. The mechanisms determining the importance of the NS80 one coiled region (aa 550–742) in aggregation and localization in the nucleus needs further research.

**Table 1:** Summary of N-terminal truncations of NS80.

Truncations	The remaining residues	Protein Size (kDa)	Form inclusions		Coils and intercoil			Interactions with other proteins									
			C	N	Coil 1	Inter coil	Coil 2	VP1	VP2	VP3	VP4	VP5	NS31	VP6	NS38	VP7	NS25
NS80	742	80	**•**	**○**	**√**	**√**	**√**	**+**	**+**	**+**	**+**	**−**	**−**	**+**	**+**	**−**	**−**
Δ 55	687	75.6	**•**	**○**	**√**	**√**	**√**	**+**	**+**	**+**	**+**				**−**		
Δ 85	657	72.2	**•**	**○**	**√**	**√**	**√**	**+**	**+**	**−**	**+**				**−**		
Δ 126	616	67.8	**•**	**○**	**√**	**√**	**√**	**+**	**+**	**−**	**+**				**−**		
Δ 216	526	57.8	**•**	**○**	**√**	**√**	**√**	**+**	**+**	**−**	**+**				**−**		
Δ 283	459	50.5	**•**	**○**	**√**	**√**	**√**	**+**	**+**	**−**	**+**				**−**		
Δ 373	369	40.6	**•**	**○**	**√**	**√**	**√**	**+**	**+**	**−**	**+**				**−**		
Δ 417	325	35.6	**•**	**○**	**√**	**√**	**√**	**+**	**+**	**−**	**+**				**−**		
Δ 491	251	27.6	**•**	**•**	**√**	**√**	**√**	**+**	**+**	**−**	**+**				**−**		
Δ 499	243	26.7	**•**	**•**	**√**	**√**	**√**	**+**	**+**	**−**	**+**				**−**		
Δ 504	237	26.2	**•**	**•**	**√**	**√**	**√**	**+**	**+**	**−**	**+**				**−**		
Δ 512	230	25.3	**○**	**•**	**√**	**√**	**√**	**+**	**+**	**−**	**+**				**−**		
Δ 549	193	21.2	**○**	**•**	**×**	**√**	**√**	**+**	**+**	**−**	**+**				**−**		
Δ 614	128	14.1	Diffusely		**×**	**×**	**√**										

C: cytoplasm.

N: nucleus.

•: located in the cytoplasm or nucleus.

○: not located in the cytoplasm or nucleus.

√: had coils or intercoil.

×: had no coils or intercoil.

+: interactions with other proteins.

−: no interactions with other proteins.

blank grid: not tested.

By sequence comparison and intracellular localization studies, a conserved region near the NS38 N-terminal was identified, which was important for the interactions between NS38 and NS80. This region is conserved in aquareoviruses, which hints that it could have a common role in aquareovirus factories formation. Nonstructural proteins encoded by S4 and S9 genome segments are important components in aquareovirus factories formation. It is similar to the formation of viral factories in orthoreoviruses. In MRV and ARV, viral factories are formed by nonstructural proteins μNS and σNS [36]–[40].

NS80 recruited not only nonstructural proteins but also structural proteins such as VP1, VP2, VP3, and VP4. These four proteins are viral inner core proteins. They also function as viral RNA dependent RNA polymerases, helicases, and GTPases that are needed in viral replication [41]. The fact that NS80 could completely colocalize with VP1, VP2, VP3, or VP4 reveals that its interactions with the enzymatic proteins is the strongest among the examined structural proteins. It indicates that NS80 has functions as the organization center in viral factories formation. NS80 aggregates to form the framework with NS38 and then recruits other viral proteins to participate in virus replication. The viral inclusions like structures formed by MRV μNS could also recruit viral core surface proteins [42], [43]. Interestingly, NS80 truncations (NS80-Δ512, NS80-Δ549) recruited structural proteins (VP1, VP2, and VP4) to the nucleus. This phenomenon was not reported in other reoviruses, which indicated the importance of NS80 N-terminal residues and the difference between aquareovirus and other reoviruses.

The NS80 residues required for interactions with other viral proteins (VP1, VP2, VP3, VP4, and NS38) were also examined (Fig. 6). Unlike MRV μNS, which recruits only one protein λ3 via the C-terminal region [43], NS80 recruits three proteins (VP1, VP2, and VP4) via the C-terminal region. It is difficult to accurately identify the NS80 residues responsible for interactions with VP1, VP2, or VP4. Results also showed that NS80 recruited NS38 and VP3 through different N-terminal regions. Locations of these functional domains indicate the difference between aquareovirus and other reoviruses.

**Figure 7:**
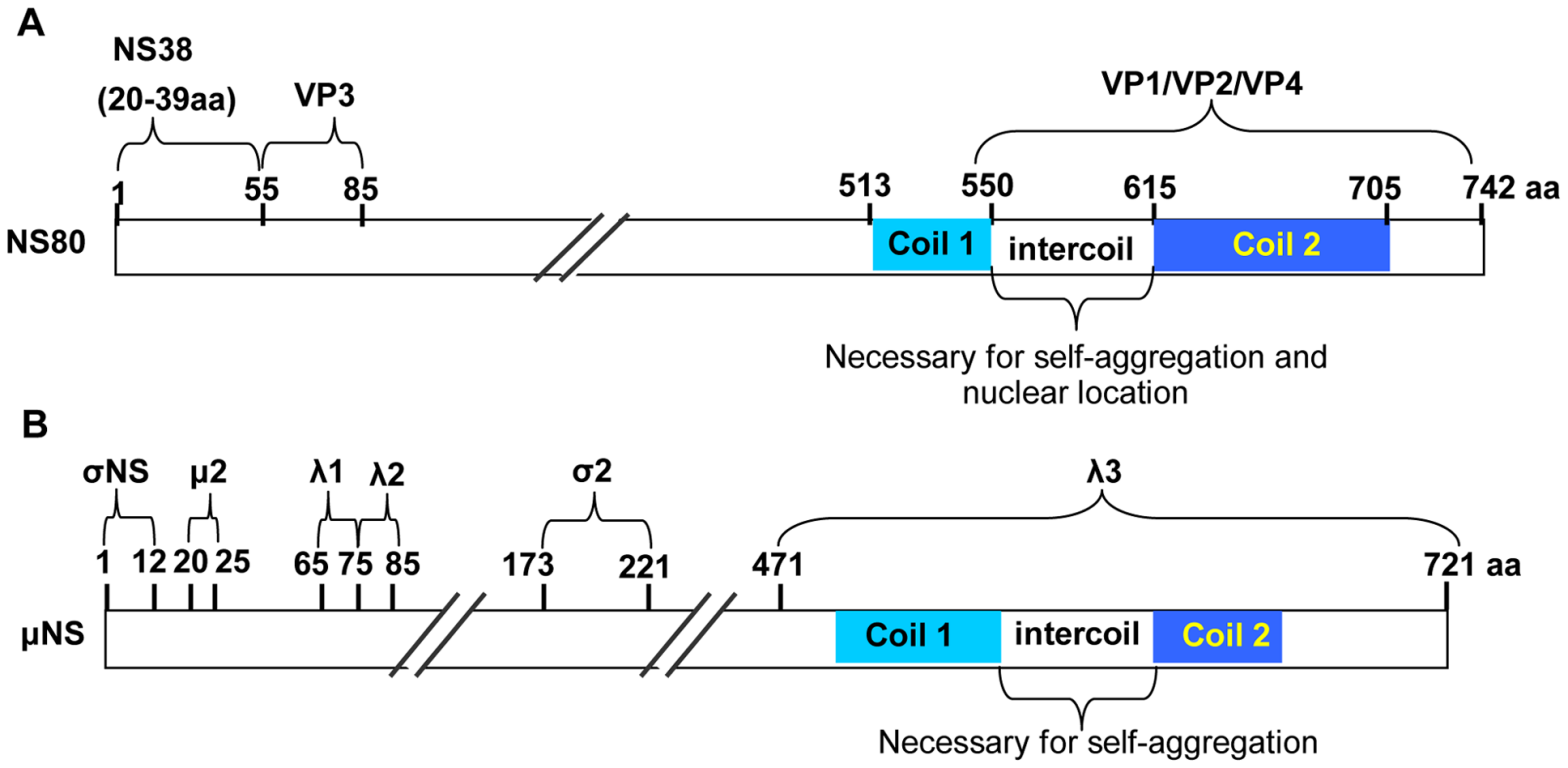
Diagram of NS80 and μNS regions required for protein interactions. A. Diagram of NS80 regions required for associations with VP1, VP2, VP3, VP4, and NS38. aa 1–55 of NS80 and aa 20–39 of NS38 were responsible for interactions with each other, and aa 56–85 of NS80 were responsible for interactions with VP3. aa that are required for associations with VP1, VP2, and VP4 belonged to regions 550–742 of NS80. The intercoil region is important for self-aggregation and nuclear localization. B. Diagram of MRV μNS regions required for interactions with λ1, λ2, λ3, μ2, σ2, and σNS. aa 1–12 of μNS were responsible for interactions with σNS. aa 20–25 were responsible for interactions with μ2. aa 65–75 and aa 75–85 were responsible for interactions with λ1 and λ2. aa 173–221 were responsible for interactions with σ2, and aa 471–721 were required for interactions with λ3. The intercoil region of μNS is important in self-aggregation.

Aquareovirus and orthoreovirus are considered as the most closely related genera in the family Reoviridae. Most of their proteins share a similarity of more than 20% (Table 2). Similarity levels of more than 20% in aa have been considered as an indication of common ancestry within a family [5], [6]. The key proteins for viral factories formation in these two viruses are nonstructural proteins (NS80 for GCRV, μNS for MRV). However, there are some differences in protein recruitment in viral factories formation. For example, the similarity shared by GCRV VP1/MRV λ2 and GCRV VP4/MRV μ2 are 28% and 27%, respectively (Table 2). The residual regions of μNS responsible for recruiting λ2 and μ2 are located in the N-terminal (aa 75–85 and 20–25, respectively; Fig. 7B), but the NS80 residual regions responsible for recruiting VP1 and VP4 are located at the C-terminal end (Fig. 7A). Another difference is the minimal region needed for viral factories formation. These differences indicated the divergence between aquareovirus and orthoreovirus.

**Table 2:** Comparison of coding proteins between GCRV and MRV.

Genome segment (encoded protein)		Amino acids similarity (%)
GCRV	MRV	
S1(VP1)	S2(λ2)	28
S2(VP2)	S1(λ3)	42
S3(VP3)	S3(λ1)	34
S4(NS80)	S6(μNS)	20
S5(VP4)	S4(μ2)	27
S6(VP5)	S5(μ1, μ1N/C)	26
S8(VP6)	S8(σ2)	25
S9(NS38)	S9(σNS)	24

The MRV μNS-based platforms have been established in animal cells and yeast for visualizing protein interactions [44], [45]. Recently, μNS of ARV has also been used for visualizing protein interactions [40], [46]. An additional nuclear localization sequence was fused to the ARV μNS so that it could work in the nucleus. In the present study, VFLS formed by the NS80 C-terminal located in the nucleus making it possible for the NS80-based protein interaction platforms to work directly in the nucleus.

Interestingly, there were at least two bands detected in Western blot analysis of NS80 expression in infected cells (Fig. 3B). The NS80 polymorphism may be because of in-frame translation. More than 20 in-frame AUG codons were found in NS80, many of which were in accordance with the Kozak's initiation consensus sequence [47]. There are two forms of μNS in MRV infected cells, μNS and μNSC. Translation of μNSC starts from an in-frame AUG codon of M3 genome segment and lacks the first 40 N-terminal residues of μNS [48]. Another possible mechanism of the polymorphism is post-translational modification. Avian orthoreovirus (ARV, another orthoreovirus) generates μNS and μNSC by post-translational cleavage [49]. The exact mechanism of NS80 polymorphism needs to be further investigated. The NS80 polymorphism may be critical to the formation of viral factories. Different NS80 lengths could meet different needs in viral infection. It could increase the specificity and efficiency of recruiting different viral proteins. Understanding NS80 could improve our knowledge about the mechanisms involved in the formation of viral factories of aquareovirus and the mechanism of antiviral research in aquaculture animals [50], [51].

In conclusion, the present study shows the detailed interactions between NS80 and NS38 or other viral proteins. NS80 sequence and structure characteristics ensure its self-aggregation to form VFLS (either in the cytoplasm or nucleus) and recruitment of virus structural or nonstructural proteins (Summary in Table 1 and Fig. 7A). It would give us new insights into the mechanisms of viral factories formation and pathogenesis of aquareoviruses.

## Materials and Methods

### Cells and virus

Grass carp ovaries (GCO) cell lines maintained in our laboratory were used for viral infection and transfection [52]. Cells were cultured in 199 medium supplemented with 10% fetal bovine serum at 25°C and placed in CO_2_-free system. Grass carp reovirus (GCRV) used in this study was maintained in our laboratory [8].

### Plasmid constructions

Primers were designed according to GCRV sequences (Table S1).

To express a C-terminal FLAG-tagged version of NS80, GCRV genomic dsRNA was used as a template. Primers 3.1-NS80-F/R was used in reverse transcription PCR (RT-PCR). In brief, genomic dsRNA was extracted from purified viral particles using the commercial TRIzol reagent (Invitrogen), according to the protocol described by the manufacturer. cDNA was reverse transcribed in a cDNA reaction containing 5 μl 5× reaction buffer, 1 μl dNTP mix (10 mM), 1 μl recombinant ribonuclease inhibitor (20 U/μl), 1 μl M-MLV RTase (Promega). The reaction was incubated in a thermal cycler at 42°C for 1 h followed by 80°C for 5 min. PCR was performed using TaKaRa Ex Taq (TaKaRa) with the same primers. The PCR product was digested with *EcoRI*/*BamHI* and then ligated to pcDNA3.1 vector (Invitrogen) with the same enzyme treatment. The resulted plasmid was named pcDNA3.1/NS80-FLAG. The aa sequence of FLAG tag in this study was DYKDDDDK.

To generate plasmids expressing NS80 truncations with a C-terminal FLAG tag, the start codons and restriction sites were introduced at different positions in the GCRV S4 segment by PCR amplification using pcDNA3.1/NS80-FLAG as the template. Each PCR product was digested with *EcoRI*/*BamHI* and then ligated to pcDNA3.1, which had been digested with the same enzymes.

To express other GCRV proteins that fused with a C-terminal HA tag, genomic dsRNA was used as the template. RT-PCR was performed with corresponding primers of each segment. The PCR product was digested and ligated to pcDNA3.1 vector with the help of different restriction enzymes. The aa sequence of HA tag in this study was YPYDVPDYA.

To generate plasmid pcDNA3.1/NS80-H569Q-FLAG, primers 3.1-NS80-H569Q-F/R that contained mutations were used in an overlap extension PCR. Primers, enzymes, and the resulted plasmids are mentioned in Table S1. All constructed plasmids were confirmed by restriction digestion and DNA sequencing.

### Transfection and cell staining

GCO cells were seeded onto coverslips in six-well plates before transfection. Plasmids were transfected or cotransfected into GCO cells using Lipofectamine reagent (Invitrogen) according to the manufacturer's instructions. In brief, a total of 4 µg plasmid DNA was mixed with 10 µl Lipofectamine in serum free medium. After 20-min incubation, the mixture was added to cells and incubated at 25°C. The serum free medium was replaced with 199 medium supplemented with 5% fetal bovine serum after 6 h incubation. The cells were then incubated and processed for Western blotting or immunostaining.

### Prokaryotic expression, protein purification and antibody preparation

For prokaryotic NS80 and NS38 expression, fragments encoding NS80 C-terminal 342 aa and whole NS38 were amplified using primers (Table S1). The PCR products were ligated into the prokaryotic vector pET-32a or pET-28a (Novagen). The resulted recombinant plasmids, named pET32a-NS80 and pET28a-NS38, were transformed into *E. coli* BL21 (DE3). To express the fused protein, the bacteria were induced with 1 mM IPTG for 6 h at 37°C. The fused protein was purified using HisBind purification kit (Novagen), mixed with an equal volume of Freund's adjuvant (Sigma), and thereafter used to immunize rabbit (NS80) or mice (NS38). Anti-NS80 serum was collected after immunizing the rabbit thrice, and anti-NS38 serum was collected after immunizing mice five times.

Animal experimental procedures were conducted under the institutional guidelines of the Hubei province. The protocol was approved by the Committee of Wuhan University Center for Animal Experiment (Permit Number: SCXK 2008-0004). Surgery was performed under the anesthetic sodium pentobarbital, and all efforts were made to minimize animal suffering.

### Western blotting analysis

Plasmids transfected GCO cells for NS80 or NS38 expression were collected and subjected to Western blot analysis as described previously [53]. Anti-FLAG antibodies (for NS80) and anti-HA antibodies (for NS38) were used as the primary antibody at a 1∶1000 dilution followed by alkaline phosphatase-conjugated goat anti-mouse IgG (H+L) antibody at a 1∶1000 dilution (Vector laboratories Inc) as the secondary antibody. GCO cells infected with GCRV at an m.o.i of 0.1 or mock infected were collected and analyzed by Western blotting as described above.

### Immunofluorescence microscopy observation

For immunofluorescence localization, all experiments were performed according to a previous study [53]. After 24 h incubation, transfected or infected cells were fixed with 4% paraformaldehyde for 30 min. Fixed cells were permeabilized with 0.2% Triton X-100 and then blocked in 10% normal goat serum at room temperature for 1 h. The cells were incubated with primary antibodies in 1% normal goat serum for 2 h, rinsed three times for 10 min each with phosphate buffered saline (PBS) containing 1% normal goat serum, and then incubated with secondary antibodies. Anti-FLAG antibodies (mouse anti-FLAG antibody, Santa Cruz) were used for detecting NS80-FLAG recombinant protein. Anti-HA antibodies (rabbit anti-HA antibody, Santa Cruz) were used for detecting proteins that fused with HA tag. Anti-NS80 or anti-NS38 antiserum was used as the primary antibody in infected cells at a 1∶100 dilution. Alexa-546-conjugated goat anti-rabbit IgG or Alexa-488-conjugated goat anti-mouse IgG at a 1∶500 dilution (Invitrogen) was used as the secondary antibody. Hoechst 33342 staining was used to visualize the nucleus. The samples were examined under a Leica DM IRB fluorescence microscope (Leica microsystems Ltd.). The images were processed with Adobe Photoshop (Adobe Systems, CA).

### Sequence comparisons

The nonredundant protein sequence database of the National Center for Biotechnology Information (National Institutes of Health, MD, USA) was searched using BLASTP and iterative searches were performed using PSI-BLAST [54]. Multiple sequence alignments were constructed using CLUSTAL_X v1.83 and edited using GeneDoc. The GenBank accession numbers of NS38 and their homolog sequences in aquareoviruses that were compared in the present study are as follows: GCRV (AAM92741), SMReV (ADZ31985.1), Chum salmon reovirus (CHSRV, AF418302), Golden shiner reovirus (GSRV, NP_938069), American grass carp reovirus (AGCRV, YP_001837103), Grass carp reovirus HZ08 (ADJ75343.1), MRV (M18389.1) and ARV (AAG44973.1).

## Supporting Information

Table S1 **Oligonucleotide primers used in this study.**
(DOC)
