# Supplementary material for: Nonstructural Protein NS80 Is Crucial in Recruiting Viral Components to Form Aquareoviral Factories
Source: PLoS One. 2013 May 6;8(5):e63737. doi: 10.1371/journal.pone.0063737 (PMC3646018; doi:10.1371/journal.pone.0063737)
Supplement: Table S1 — Oligonucleotide primers used in this study. (DOC) [file pone.0063737.s001.doc]

Table S1 Oligonucleotide primers used in this study

| **Primer** | **Primer sequence (5’-3’)** | **Constructed**  **plasmid** |
| --- | --- | --- |
| 32a-NS80-F | 5’ TTCGAATTCCGTCACAAGCTGAC 3’ (EcoR I) | pET32a-NS80 |
| 32a-NS80-R | 5’ ACAGCGGCCGCACAGCAGCAGGGAG 3’ (Not I) |
| 28a-NS38-F | 5’ ACAGAATTCATGGCACACACAGG 3’ (EcoR I) | pET28a-NS38 |
| 28a-NS38-R | 5’ CACCTCGAGACATACCCCCGATC 3’ (XhoI) |
| 3.1-NS80-F | 5’ CTTGGATCCTAACATGGCACGCC 3’ (BamH I) | pcDNA3.1/NS80-FLAG |
| 3.1-NS80-R | 5’ ACGGAATTCTTACTTATCGTCGTCATCCTTGTAATCCAGCAGCAGGGAGGC 3’ (EcoR I) |
| 3.1-NS80-Δ55F | 5’ CAAGGATCCACAAGATGGTTTTCG 3’ (BamH I) | pcDNA3.1/NS80-Δ55-FLAG |
| 3.1-NS80-Δ85F | 5’ CAAGGATCCACAAGATGGTTTTCG 3’ (BamH I) | pcDNA3.1/NS80-Δ85-FLAG |
| 3.1-NS80-Δ126F | 5’ AACGGATCCGAAATGGTGCTTCTGCGC 3’ (BamH I) | pcDNA3.1/NS80-Δ126-FLAG |
| 3.1-NS80-Δ216F | 5’ AGCGGATCCGACGATGGCACGCG 3’ (BamH I) | pcDNA3.1/NS80-Δ216-FLAG |
| 3.1-NS80-Δ283F | 5’ TGCGGATCCTGCGATGGGTGTC 3’ (BamH I) | pcDNA3.1/NS80-Δ283-FLAG |
| 3.1-NS80-Δ373F | 5’ TCTGGATCCCATCATGGCTTGTC 3’ (BamH I) | pcDNA3.1/NS80-Δ373-FLAG |
| 3.1-NS80-Δ417F | 5’ TCCGGATCCTTGTCATGGGTATC 3’ (BamH I) | pcDNA3.1/NS80-Δ417-FLAG |
| 3.1-NS80-Δ491F | 5’ ACGGGATCCACCATGGCTGACTCCATCCAC 3’ (BamH I) | pcDNA3.1/NS80-Δ491-FLAG |
| 3.1-NS80-Δ499F | 5’ CACGGATCCATCATGGAAGCCGACAAG 3’ (BamH I) | pcDNA3.1/NS80-Δ499-FLAG |
| 3.1-NS80-Δ504F | 5’ AAGCGGATCCGGGAATGGGCGTCGC 3’ (BamH I) | pcDNA3.1/NS80-Δ504-FLAG |
| 3.1-NS80-Δ512F | 5’ GTTGGATCCACTATGGCTTCCTCCACCCTACG 3’ (BamH I) | pcDNA3.1/NS80-Δ512-FLAG |
| 3.1-NS80-Δ549F | 5’ ATCGGATCCGTCATGGCTTATACCAACCATC 3’ (BamH I) | pcDNA3.1/NS80-Δ549-FLAG |
| 3.1-NS80-Δ614F | 5’ GATGGATCCACTATGGCTCTCACCGCCCAG 3’ (BamH I) | pcDNA3.1/NS80-Δ614-FLAG |
| 3.1-NS80-H569Q-F | 5’ TACCTCCACTCCCAAACGTGCGTCAATACC 3’ | pcDNA3.1/NS80-H569Q-FLAG |
| 3.1-NS80-H569Q-R | 5’ GTATTGACGCACGTTTGGGAGTGGAGGTAC 3’ |
| 3.1-VP1-F | 5’ AAAGGATCCTGCATTATGGCTGCGG 3’ (BamHI) | pcDNA3.1/VP1-HA |
| 3.1-VP1-R | 5’ AATGAATTCTTAAGCGTAATCTGGAACATCGTATGGGTACTCAATCACGTATTC 3’ (EcoR I) |
| 3.1-VP2-F | 5’ AAAGGTACCTGTACCATGGAGGAA’ (Kpn I) | pcDNA3.1/VP2-HA |
| 3.1-VP2-R | 5’ TGAGAATTCTTAAGCGTAATCTGGAACATCGTATGGGTAAACATCACGCATC’ (EcoR I) |
| 3.1-VP3-F | 5’ TTTAAGCTTTCCACCATGCCGCG 3’ (Hind III) | pcDNA3.1/VP3-HA |
| 3.1-VP3-R | 5’ ACCCTCGAGTTAAGCGTAATCTGGAACATCGTATGGGTACGTCGCGCTGCGCA 3’ (Xho I) |
| 3.1-VP4-F | 5’ TTGGCTAGCAGGATGGTCACCATTG 3’ (Nhe I) | pcDNA3.1/VP4-HA |
| 3.1-VP4-R | 5’ TGAGGTACCTCAAGCGTAATCTGGAACATCGTATGGGTAAACCCCGGTCGAG 3’ (Kpn I) |
| 3.1-VP5-F | 5’ CGCGCTAGCTCTACAATGGGGAAC 3’ (Nhe I) | pcDNA3.1/VP5-HA |
| 3.1-VP5-R | 5’ TCACTCGAGTTAAGCGTAATCTGGAACATCGTATGGGTACTTGCCGGGCCAC 3’ (Xho I) |
| 3.1-NS31-F | 5’ ATCGAATTCTCTAACATGAACGC 3’ (EcoRI) | pcDNA3.1/NS31-HA |
| 3.1-NS31-R | 5’ CGACTCGAGTTAAGCGTAATCTGGAACATCGTATGGGTAGCAACCATTGTC 3’ (Xho I) |
| 3.1-VP6-F | 5’ AAGGGATCCTGTGATGGCACAGCG 3’ (BamH I) | pcDNA3.1/VP6-HA |
| 3.1-VP6-R | 5’ GTGGAATTCTTAAGCGTAATCTGGAACATCGTATGGGTAGACGAACATCGCCT 3’ (EcoR I) |
| 3.1-NS38-F | 5’ ATTGAATTCCATCATGGCACAC 3’(EcoR I) | pcDNA3.1/NS38-HA |
| 3.1-NS38-R | 5’ ATACTCGAGTTAAGCGTAATCTGGAACATCGTATGGGTACATACCCCCGATC 3’(Xho I) |
| 3.1-NS38-Δ19F | 5’ CGCGAATTCGGAATGGTCCGCCTCC 3’ (EcoR I) | pcDNA3.1/NS38-Δ19-HA |
| 3.1-NS38-Δ39F | 5’ CAGGAATTCGCTATGGGTGATCTTG 3’ (EcoR I) | pcDNA3.1/NS38-Δ39-HA |
| 3.1-VP7-F | 5’ GATGGATCCCACGATGCCACTTC 3’ (BamH I) | pcDNA3.1/VP7-HA |
| 3.1-VP7-R | 5’ GACGAATTCTTAAGCGTAATCTGGAACATCGTATGGGTAATCGGATGGCTCC 3’ (EcoR I) |
| 3.1-NS25-F | 5’ CCTTGGATCCCAAACATGGCCAC 3’ (BamH I) | pcDNA3.1/NS25-HA |
| 3.1-NS25-R | 5’ AGAGAATTCTTAAGCGTAATCTGGAACATCGTATGGGTATTCAAACACGCA 3’ (EcoR I) |
